# Consensus scoring-guided virtual screening identifies potent anti-saprolegniasis compounds targeting a P450 fusion protein

**DOI:** 10.3389/fmicb.2025.1723326

**Published:** 2026-01-21

**Authors:** Muhammad Akhtar Ali, Anum Javaid, Charuvaka Muvva, Natarajan Arul Murugan, Vaibhav Srivastava

**Affiliations:** 1 Division of Glycoscience, Department of Chemistry, CBH School, Royal Institute of Technology (KTH), AlbaNova University Centre, Stockholm, Sweden; 2 School of Biological Sciences, University of the Punjab, Lahore, Pakistan; 3 Council of Scientific and Industrial Research–Central Leather Research Institute (CSIR–CLRI), Chennai, India; 4 Department of Computational Biology, Indraprastha Institute of Information Technology, New Delhi, India

**Keywords:** anti-saprolegnia compounds, consensus screening, P450 fusion protein, saprolegniasis, virtual screening

## Abstract

Saprolegniasis, predominantly caused by *Saprolegnia* spp., particularly *Saprolegnia parasitica*, has reemerged as a major threat in aquaculture, resulting in substantial economic losses of millions of dollars annually. Historically, malachite green was highly effective against this disease; however, its use was banned in aquaculture due to its carcinogenic nature. Consequently, there is an urgent need for novel and effective agents to mitigate economic losses. Several studies, including a subtractive-proteomics study from our laboratory, have identified multiple anti-saprolegnia compounds; however, their efficacy remains to be confirmed *in vivo*. Targeting mitochondrial energy production in *Saprolegnia* offers a potential strategy to combat this pathogen. Notably, cytochrome P450 is unique to *Saprolegnia* species and was previously shown to be inhibited by malachite green. In this study, we performed a virtual screening of FDA-approved drugs to identify compounds that target P450 and thereby disrupt energy production. To ensure robust ranking of potential inhibitors, we integrated multiple docking tools and applied consensus scoring. Based on ranking and water solubility, selected compounds were subjected to *in vitro* testing. Among these, chlorhexidine and diminazene exhibited strong anti-saprolegnia activity in liquid culture, with MIC₅₀ values of 10.93 and 417 μg/ml, respectively. Although chlorhexidine was less potent than malachite green, it demonstrated substantial inhibitory activity at low microgram per milliliter concentrations, highlighting its potential as a promising candidate for further development in aquaculture.

## 1 Introduction

The agriculture and aquaculture sectors suffer multi-billion-dollar losses inflicted by a fungal-like eukaryotic group of organisms called oomycetes, also known as water moulds (Baldauf et al., 2000; van West, 2006; Diéguez-Uribeondo et al., 2009; Beakes et al., 2012; Balci and Bienapfl, 2013). The notorious Irish potato famine was caused by late blight, a disease brought on by the oomycete *Phytophthora infestans*, resulting in a human dilemma and mass migration (Davison, 1998). The aquatic oomycetes, such as *Saprolegnia* species, are creating devastating havoc in the aquaculture industry by causing ultimately lethal infections in various farmed and wild fishes, including salmon, tilapia and catfish (van West, 2006). According to the State of World Fisheries and Aquaculture (SOFIA) 2024 report by the Food and Agriculture Organisation (FAO), approximately 62 million people worldwide earn their livelihoods in fisheries and aquaculture, highlighting the critical socio-economic importance of controlling these pathogens (Food and Agriculture Organization, 2024).

The oomycete pathogens are a devastating threat to global food security, biological diversity and the ecosystem. Oomycetes possess a range of abilities that make them particularly challenging to control. These include their capacity to modulate host processes (Schornack et al., 2009; Bozkurt et al., 2012), diverse reproductive strategies such as sexual, asexual, and interspecific hybridisation (Thines, 2014), rapid proliferation that can offset limited recombination, bipartite genomes with both fast- and slow-evolving regions, polyploidy, and the ability to jump to new hosts, all of which contribute to their continuously and rapidly evolving fitness.

Saprolegniasis, often referred to as winter disease or cotton wool disease in aquaculture, is caused by Saprolegnia species in fish and amphibians, leading to extensive tissue damage and multimillion-dollar losses annually in aquaculture (Molina et al., 1995; van West, 2006; Berg et al., 2013; Derevnina et al., 2016). Previously, saprolegniasis was controlled using malachite green, which later proved to be carcinogenic and was consequently banned in the aquaculture industry (National Toxicology Program, 2005). Recent studies have identified several new compounds that inhibit *Saprolegnia* growth *in vitro* through subtractive proteomics and molecular docking approaches for target identification and virtual drug screening (Kumar et al., 2020; Dehkordi et al., 2025; Tang et al., 2025; Tippayakraisri et al., 2025). However, their efficacy *in vivo* remains to be determined, highlighting the urgent need for novel, potent therapeutics to control this devastating disease.

Heme-thiolate proteins such as cytochrome P450 are present across all domains of life, from viruses to humans, and play critical roles in primary and secondary metabolism due to their monooxygenase activity (Lamb et al., 2009; Makishima, 2014). Their dynamic nature and stereo- and regio-specific enzymatic activity make them valuable/essential enzymes for living organisms, especially in microorganisms, including fungi, and the biotechnological industry. They are also highly promising alternative drug targets in various domains of life due to their role in sterol biosynthesis (Kelly and Kelly, 2013; Jawallapersand et al., 2014). After the discovery of the first P450 fusion protein in *Bacillus megaterium* (Ruettinger et al., 1989), several P450 fusion proteins with redox or other proteins have been described in various life forms. In oomycetes, a unique P450 fusion protein belonging to the CYP5619 family, particularly CYP5619A1, has been identified in the fish pathogens *Saprolegnia diclina* and *Saprolegnia parasitica*. Molecular docking studies have demonstrated that malachite green, a highly potent inhibitor of saprolegniasis, binds strongly to CYP5619A1 (Bamal et al., 2018). We hypothesise that this high affinity underlies the efficacy of malachite green, and that CYP5619A1 represents a promising drug target for identifying similarly potent, non-carcinogenic molecules to combat saprolegniasis and other oomycete diseases in animals and plants.

In this study, we performed virtual screening of FDA-approved drugs to identify compounds targeting CYP5619A1 that is a P450–reductase fusion protein unique only to *Saprolegnia* spp. We integrated multiple molecular docking tools—Autodock 4.0, Autodock Vina, and Dock6—followed by consensus scoring to improve the reliability of lead compound selection (Feher, 2006; Houston and Walkinshaw, 2013; Masters et al., 2020). Candidate compounds were further evaluated for physicochemical properties, including water solubility, to prioritise molecules suitable for practical application. By integrating computational predictions with experimental validation, this approach aims to identify safe and effective inhibitors targeting unique CYP5619A1 fusion protein, bridging the gap between *in silico* discovery and therapeutically relevant anti-saprolegnia agents for the aquaculture industry.

## 2 Results

### 2.1 P450 structure modelling and *in silico* drug screen

**Figure 1:**
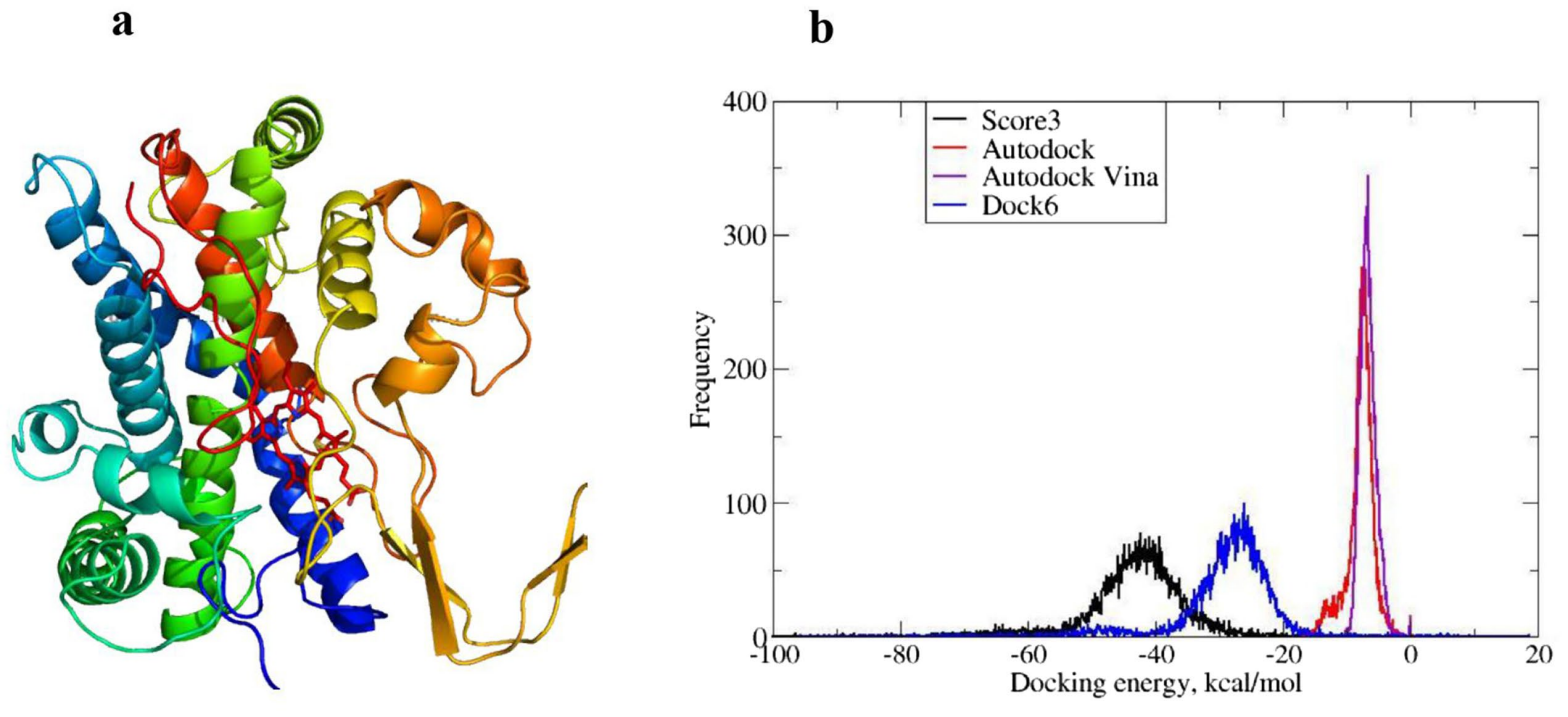
P450 and the docking energies from molecular docking of FDA-approved drugs against it. **(a)** The modelled protein structure of CYP5619A1. **(b)** The frequency and docking energy distribution of hit compounds from various molecular docking tools, along with the cumulative scoring function (Score3), are shown.

The Modeller software (Sali and Blundell, 1994) was used to build a 3D model of the CYP5619A1’s P450 domain. Among all templates, CYP120A1 (PDB ID: 2VE3) (Kühnel et al., 2008) showed the lowest E-value and was therefore selected as the template to build the 3D model of CYP5619A1. The modelled structure is shown in Figure 1a. The HEME group was considered during the structure modelling.

The drug screening strategy involved the molecular docking of the FDA-approved drugs from DrugBank (DB) against the P450. The binding site information was based on known information about the binding site amino acid residues, which were shown to interact with malachite green. Three different tools—AutoDock4.0, AutoDock Vina, and Dock6—were used to screen the compounds individually. Most hit compounds revealed by AutoDock4.0 and AutoDock Vina have relatively comparable docking energies, which are higher than the majority of hit compounds found by using Dock6 (Figure 1a). Additionally, the cumulative docking energy was defined as the sum of docking energies from all three docking tools. The compounds in this list are reordered and show lower docking energies (Supplementary Table S1) compared to their individual docking energies from each docking tool (Figure 1b). Figure 1b shows the distribution of the docking energies as computed from the aforementioned molecular docking software.

Additionally, it displays the distribution of the new scoring function, which is the sum of the three individual scoring functions. It is worth recalling that Autodock4.0 and Dock6 implement physics-based scoring functions, whereas Autodock Vina employs an empirical scoring function to rank protein-ligand complexes. The distribution of scoring functions from Autodock4.0 and Autodock Vina is comparably similar, while the Dock6 scores are underestimated. The top hundred compounds from this list were selected as the final hit compounds (Supplementary Table S1). The lowest binding energy of −111.82 kcal/mol was shown by chlorhexidine in the final list, and this was mainly contributed by binding energy (−95.82 kcal/mol) from Dock6, where it was the top hit compound (Supplementary Table S1).

The compounds to be tested *in vitro* were selected based on their: (a) water solubility, (b) availability, and (c) rank in the final list of hit compounds. The seven compounds tested for *in vitro* anti-saprolegnia activity at concentrations up to 1 mg/mL were chlorhexidine, diminazene, phosphocreatine, moroxydine, famotidine, metformin, and streptomycin. The two successful anti-saprolegnia compounds from the *in vitro* assay, along with their corresponding binding energies, are shown in Table 1.

**Table 1:** The successful anti-saprolegnia drugs and their binding energies, as determined by various molecular docking tools.

No.	Compound name	DB ID	Binding energies (kcal/mol)			
			AutoDock	AutoDock Vina	Dock6	Accumulative
1	Chlorhexidine	DB00878	−8.40	−7.60	−95.82	−111.82
2	Diminazene	DB03608	−6.98	−7.2	−71.73	−86.25
3	Malachite green	DB03895	−7.47	−7.1	−47.90	−62.47

### 2.2 Chlorhexidine

**Figure 2:**
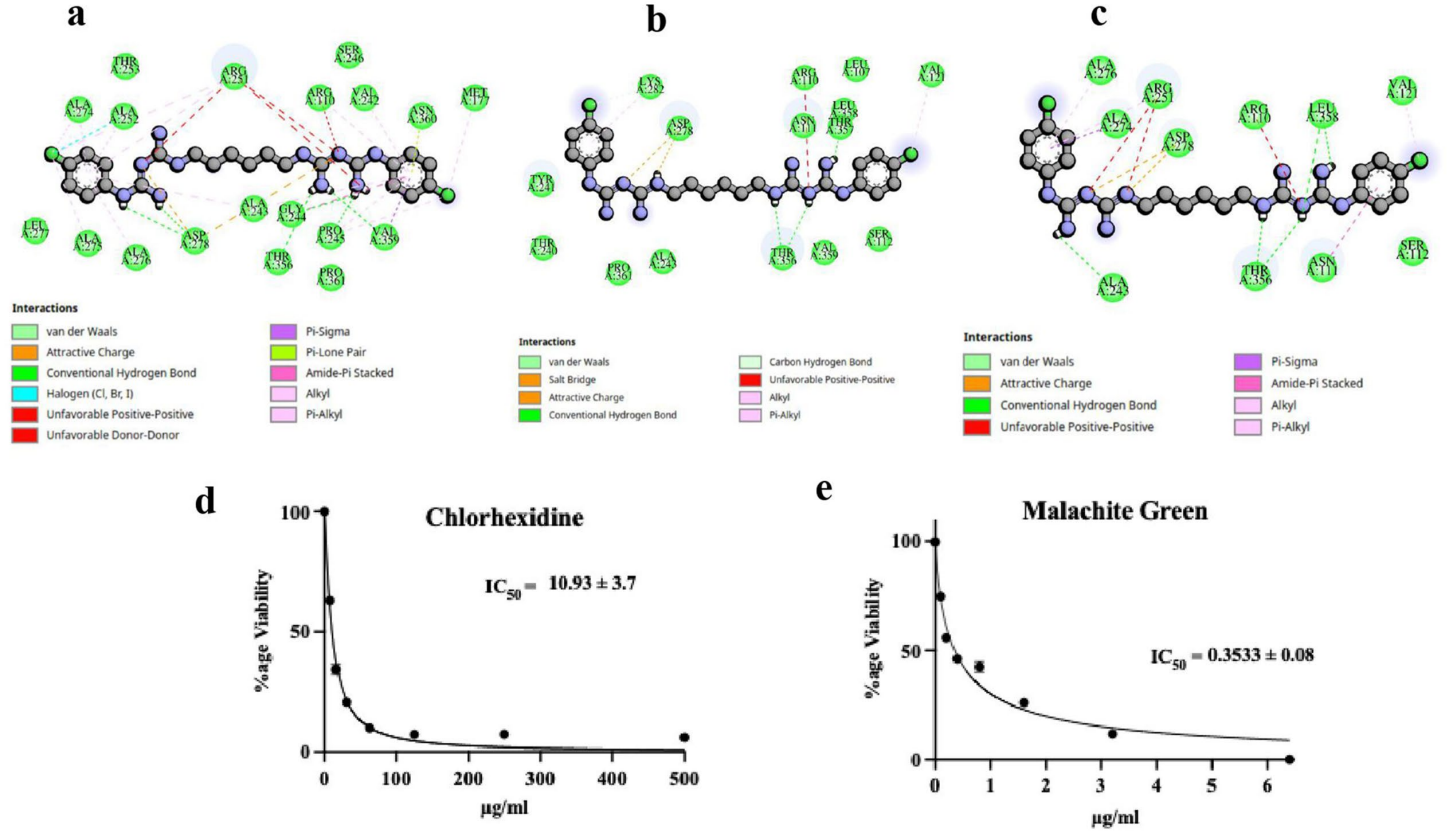
Molecular docking and *in vitro* anti-oomycetes drug dose response of chlorhexidine. The P450 interacting residues and interaction with chlorhexidine from the molecular docking studies by using **(a)** AutoDock4.0, **(b)** Dock6, and **(c)** AutoDock Vina are shown in terms of three-letter amino acid code and dotted green lines for hydrogen bonding, respectively. The MTT assay was used to determine the IC_50_ of **(d)** Chlorhexidine, and **(e)** Malachite green as a positive control.

**Figure 3:**
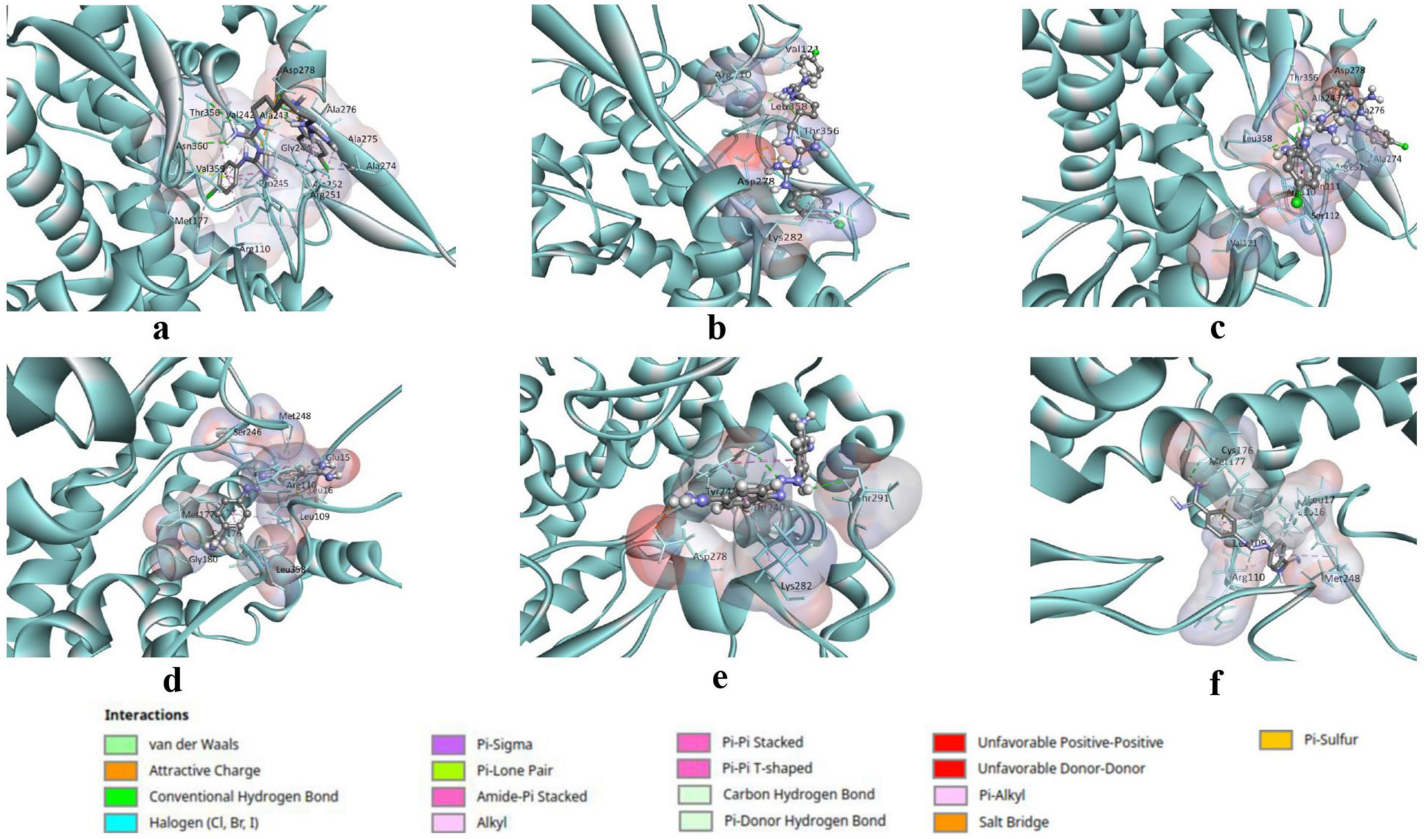
3D visualisation of the docked complexes of the P450 protein with chlorhexidine and diminazene. The P450 protein is displayed in a solid ribbon style in cyan color, while its interacting residues are shown as sticks. The interacting pocket of the P450 protein is represented as a transparent surface, coloured according to the atomic charges of the residues. The interacting amino acid residues are labelled in 3-letter and number format with black font. The P450 interacting residues and 3D interactions with chlorhexidine are depicted in **(a–c)**, where each complex is derived from docking poses from the molecular docking studies by using **(a)** AutoDock4.0, **(b)** Dock6, and **(c)** AutoDock Vina. However, the P450 interacting residues and 3D interactions with diminazene are depicted in **(d–f)**, where each complex is derived from docking poses from the molecular docking studies by using **(d)** AutoDock4.0, **(e)** Dock6, and **(f)** AutoDock Vina.

The accumulative binding energy of chlorhexidine was the lowest of all the hit compounds, and the value was −111.82 kcal/mol. The binding energy obtained from Dock6 (−95.82 kcal/mol) contributed the major proportion of this binding energy. The binding energies of chlorhexidine from AutoDock4.0 and the AutoDock Vina were −8.4 and −7.6 kcal/mol, respectively (Table 1). The interacting P450 residues with chlorhexidine when docked using AutoDock4.0 and Autodock Vina, were Arg110, Met177, Val242, Ala243, Gly244, Pro245, Arg251, Ala252, Ala274, Ala275, Ala276, Asp278, Thr356, Val359 & Asn360 and Arg110, Asn111, Val121, Ala243, Gly244, Pro245, Arg251, Ala274, Ala276, Asp278, Thr356, Leu358 & Val359, respectively (Figures 2a,c). In both cases, the binding occurs in the same binding site, which can be deduced from the involvement of the same residues, such as Arg110, Ala243, Arg251, etc. as enlisted in Table 2. However, in the case of Dock6 the interacting residues were Arg110, Val121, Asp278, Lys282, Thr356, and Leu358 (Figure 2b). The number of hydrogen bonds in the AutoDock, AutoDock Vina and Dock6 were 6, 5, and 4, respectively (Figures 2a–c). The 50% growth inhibitory concentration (IC₅₀), determined using a sodium resazurin–based MTT assay, was 10.93 μg/mL for chlorhexidine (Figure 2d) in liquid Saprolegnia cultures grown in Machlis medium, while malachite green showed an IC₅₀ of 0.3533 μg/mL (Figure 2e). The 3D interactions of the docked complexes of the P450 protein with chlorhexidine for each docking tool are shown in Figures 3a–c.

**Table 2:** P450 interacting residues, and their interaction types, as determined by various molecular docking tools.

Ligand name	Amino acid residues of P450 interact with the ligand			Interaction/s type
	AutoDock	AutoDock Vina	Dock6	
Chlorhexidine	Arg110, Met177, Val242, Ala243, Gly244, Pro245, Arg251, Ala252, Ala274, Ala275, Ala276, Asp278, Val359, and Asn360	Arg110, Asn111, Val121, Arg251, Ala274, Ala276, and Asp278	Arg110, Val121, Asp278, and Lys282	Hydrophobic interactions
	Gly244, Pro245, Asp278, Thr356, and Val359	Ala243, Thr356, and Leu358	Lys282, Thr356, and Leu358	Hydrogen bonding
Diminazene	Glu15, Leu16, Leu17, Leu109, Arg110, Cys176, Met177, Gly180, Met248, and Leu358	Leu16, Leu17, Leu109, Arg110, Cys176, Met177, and Met248	Thr240, Tyr241, Asp278, Lys282, and Thr291	Hydrophobic interactions
	Glu15 and Cys176	Arg110 and Cys176	Thr240, Tyr241, and Thr291	Hydrogen bonding

### 2.3 Diminazene

**Figure 4:**
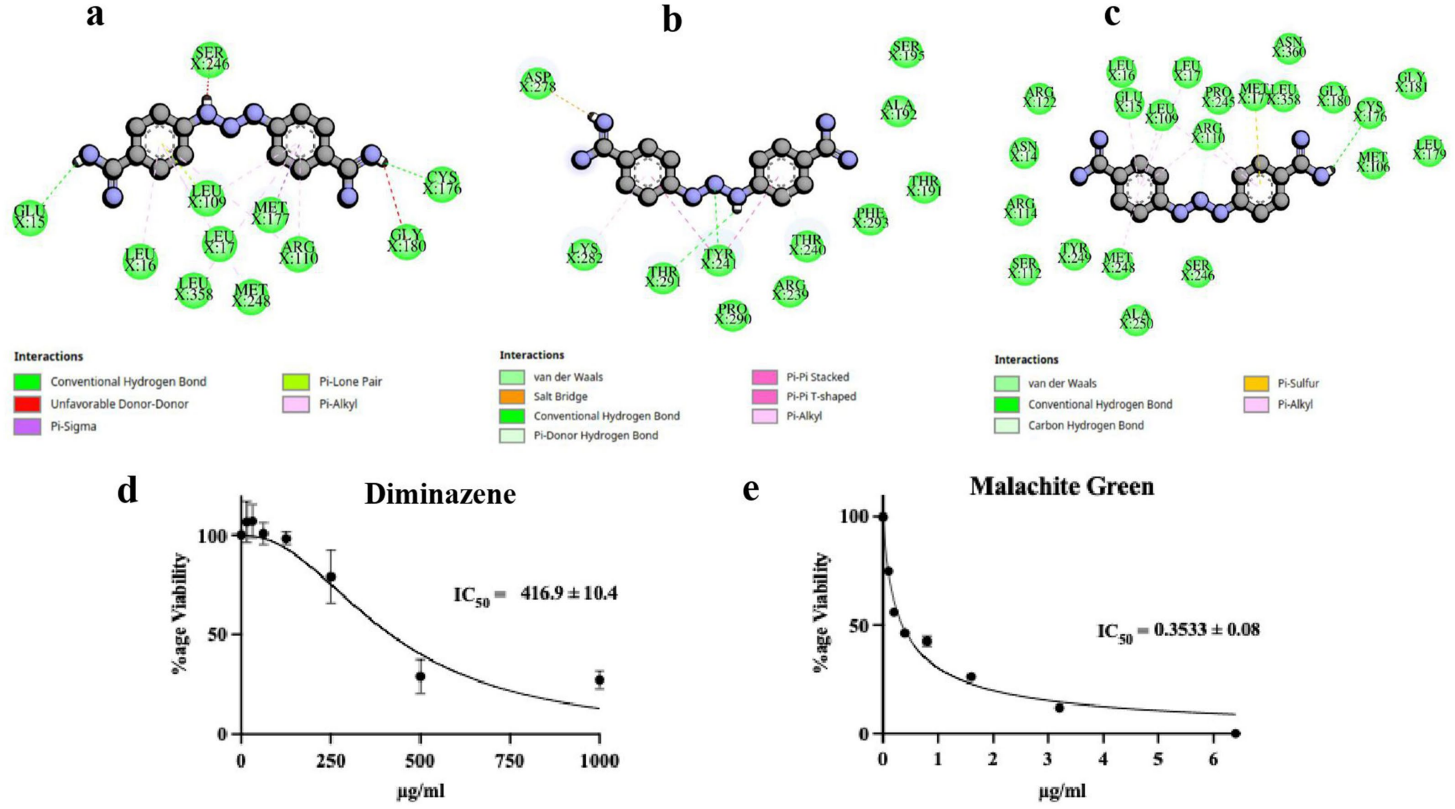
Molecular docking and *in vitro* anti-oomycetes drug dose response of diminazene. The P450 interacting residues and interaction with diminazene from the molecular docking studies by using **(a)** AutoDock, **(b)** Dock6, and **(c)** AutoDock Vina are shown in terms of three amino acid codes and dotted green lines for hydrogen bonding, respectively. The MTT assay was used to determine the IC_50_ of **(d)** diminazene and **(e)** malachite green as a positive control.

The accumulative binding energy for diminazene was −86.25 kcal/mol, and from Dock6 scoring was −71.7 kcal/mol, respectively (Table 1). The interacting P450 residues with diminazene when docked using AutoDock4.0 were Glu15, Leu16, Leu17, Leu109, Arg110, Cys176, Met177, Gly180, Met248, and Leu358 (Figure 4a). In the case of AutoDock Vina, the interacting residues were Leu16, Leu17, Leu109, Arg110, Cys176, Met177, and Met248 (Figure 4c). In both cases, the binding occurs in the same binding site, which can be deduced from the involvement of the same residues, such as Leu16, Leu17, Leu109, etc. (Table 2). For Dock6, the residues Thr240, Tyr241, Asp278, Lys282, and Thr291 were involved in overall interactions (Figure 4b). The residues involved in hydrogen bonding interactions with the ligand for each derived complex have been enlisted in Table 2. Hence, the number of hydrogen bonds in the case of AutoDock, AutoDock Vina and Dock6 was 2, 2, and 3, respectively (Figures 4a–c). As can be seen from the above discussion, different molecular docking software predict different binding poses for the ligand in the binding site. The binding conformation of chlorhexidine is shown in Figure 4a. As can be seen, AutoDock predicts a folded conformer, AutoDock Vina predicts a partially folded conformer, and Dock6 predicts a linear or extended conformer. The IC_50_ value determined for diminazene in liquid Saprolegnia culture in Machlis medium was 417 μg/mL (Figure 4d), as determined by the MTT assay. The 3D interactions of the docked complexes of the P450 protein with diminazene for each docking tool are shown in Figures 3d–f.

### 2.4 Molecular dynamics simulation

#### 2.4.1 RMSD analysis of protein-ligand complexes

**Figure 5:**
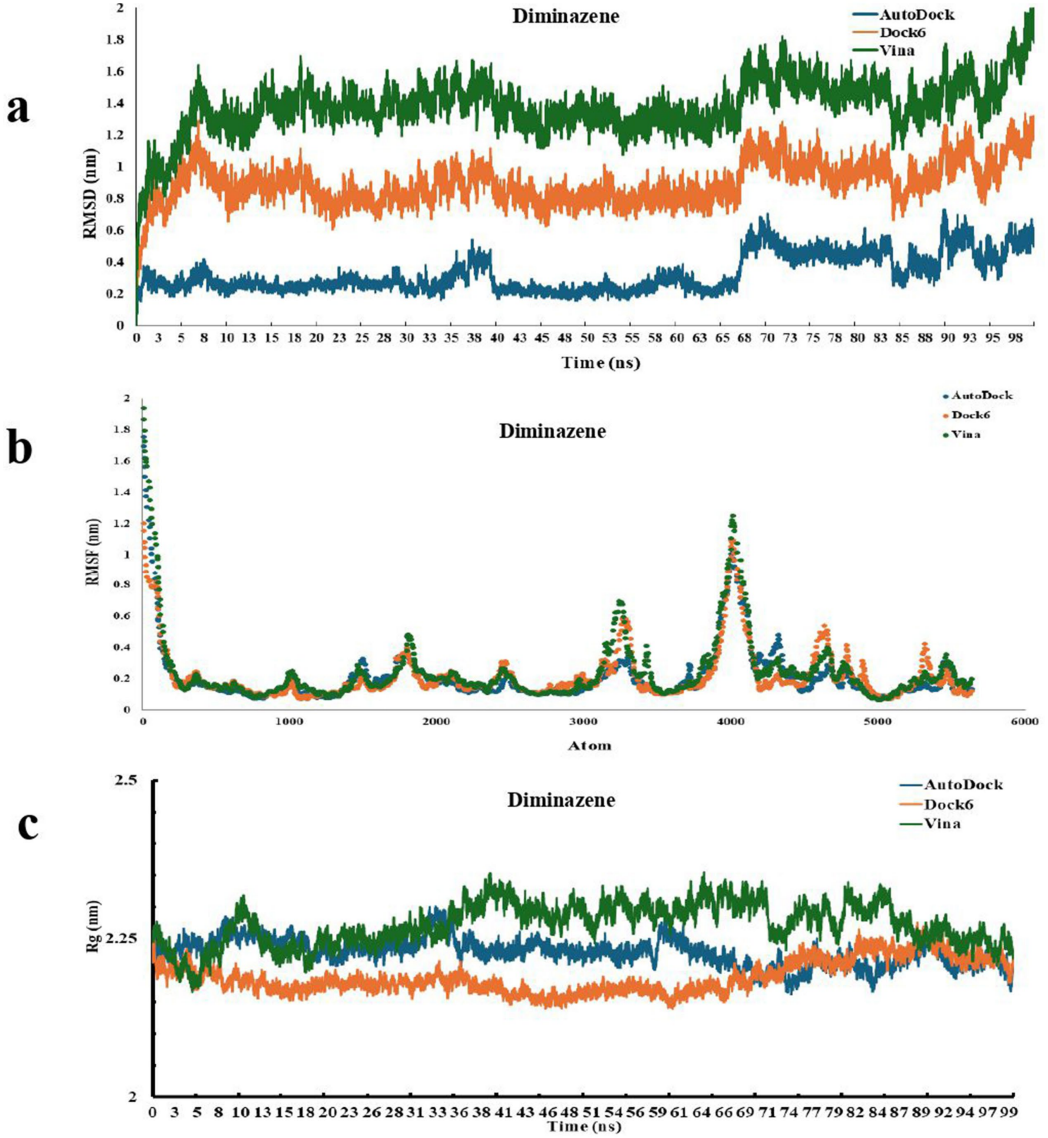
Molecular dynamics simulations of P450-diminazene complexes derived from AutoDock, Dock6, and Vina docking tools. **(a)** The root mean square deviation (RMSD) analysis of P450-diminazene complexes over a 100 ns simulation shows the structural stability of complexes formed by AutoDock (blue), Dock6 (orange), and Vina (green) docking poses. On the X-axis, time is plotted in nanoseconds (ns), and on the Y-axis, RMSD is plotted in nanometers (nm); **(b)** The root mean square fluctuation (RMSF) analysis of P450-diminazene complexes over a 100 ns simulation: indicates the residue-level flexibility of P450 protein in complexes derived from AutoDock (blue), Dock6 (orange), and Vina (green) docking poses. X-axis shows the atom of P450 protein and Y-axis shows RMSF in nanometers (nm); **(c)** Radius of gyration (Rg) plots of P450-diminazene complexes over 100 ns simulation: depict the structural compactness of complexes formed by AutoDock (blue), Dock6 (orange), and Vina (green) docking poses. Time is represented in nanoseconds (ns) on the X-axis, while Rg is shown in nanometers (nm) on the Y-axis.

**Figure 6:**
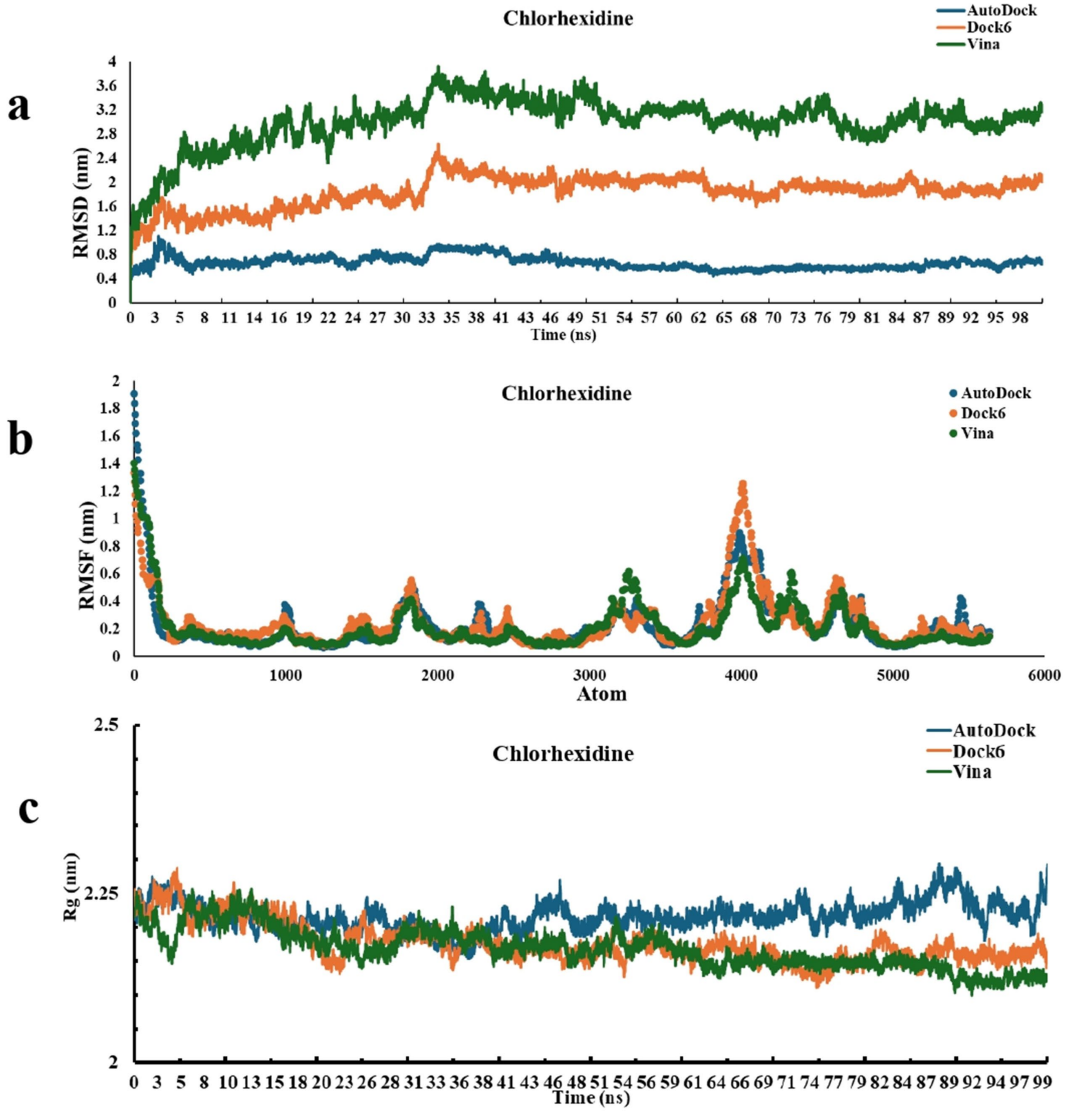
Molecular dynamics simulations of P450-chlorhexidine complexes derived from AutoDock, Dock6, and Vina docking tools. **(a)** The root mean square deviation (RMSD) analysis of P450-chlorhexidine complexes over a 100 ns simulation shows the structural stability of complexes formed by AutoDock (blue), Dock6 (orange), and Vina (green) docking poses. On the X-axis, time is plotted in nanoseconds (ns), and on the Y-axis, RMSD is plotted in nanometers (nm); **(b)** The root mean square fluctuation (RMSF) analysis of P450-chlorhexidine complexes over a 100 ns simulation: indicates the residue-level flexibility of P450 protein in complexes derived from AutoDock (blue), Dock6 (orange), and Vina (green) docking poses. X-axis shows the atom of P450 protein and Y-axis shows RMSF in nanometers (nm); **(c)** Radius of gyration (Rg) plots of P450-chlorhexidine complexes over 100 ns simulation: depict the structural compactness of complexes formed by AutoDock (blue), Dock6 (orange), and Vina (green) docking poses. On the X-axis, time in nanoseconds (ns) is represented, while on the Y-axis, Rg in nanometers (nm) is shown.

The structural stability of the P450-ligand complexes generated from AutoDock, Dock6, and Vina docking poses was determined using RMSD analysis over 100 ns simulations (Figures 5a, 6a). Within the first ~10 ns, all systems reached equilibrium and maintained equilibrated trajectories thereafter. For diminazene, the AutoDock-derived complex depicted relatively lower RMSD (0.2–0.6 nm), thus a higher stability, when compared to the RMSD values of Dock6-derived complex (0.8–1.2 nm), and Vina-derived complex (1.2–1.6 nm) which showed relatively larger fluctuations, thus suggesting more conformational changes (Figure 5a). While for chlorhexidine, the AutoDock-derived complex again showed comparatively smaller deviations and higher stability (~0.2–0.6 nm) than the Dock6-derived complex and the Vina-derived complex that stabilised around 1.0–2.0 nm, and ~2.0–3.5 nm, respectively (Figure 6a). Overall, under current simulation parameters, these findings suggest that AutoDock-derived docked poses for both ligands generated relatively stable conformations, whereas Vina-derived complexes provided greater flexibility in the protein structure.

#### 2.4.2 RMSF analysis of protein-ligand complexes

RMSF profiles were computed to assess the residual flexibility of P450-ligand complexes generated from AutoDock, Dock6, and Vina docking poses (Figures 5b, 6b). The residues in all models showed relatively low fluctuations (<0.4 nm), indicating the overall structural stability. While, relatively higher flexibility (RMSF >0.4 nm) was noted at the N-terminus and loop regions, as they are solvent-exposed segments (Martínez, 2015; Su et al., 2020; Paul et al., 2022) of the P450 protein. Additionally, diminazene and chlorhexidine reduced the fluctuations in the residues residing within and around the active site compared to the apo protein, implying that ligand binding increases the stability and rigidity of the catalytic pocket. The RMSF patterns extracted from AutoDock, Dock6, and Vina-derived complexes for both diminazene and chlorhexidine were largely similar, demonstrating that, despite considerable differences in global stability observed in RMSD, residue-level dynamics were suggested to preserve across multiple docking poses of both drugs, under simulation conditions.

#### 2.4.3 Radius of gyration (Rg) analysis

During the simulations of P450-ligand complexes generated from AutoDock, Dock6, and Vina docking poses, the radius of gyration (Rg) was also calculated to evaluate their global compactness and structural integrity (Figures 5c, 6c). In the case of Diminazene, the AutoDock and Dock6-derived complexes maintained relatively steady Rg values ranging from ~2.2 to 2.3 nm across the simulation. Still, the Vina-derived complex fluctuated slightly more (~2.25–2.35 nm), indicating a slightly increased conformational flexibility and decreased stability (Figure 5c). However, for chlorhexidine, all complexes maintained steady Rg profiles, with those derived from AutoDock reporting values around 2.2 to 2.35 nm, and those of Dock6 and Vina-derived displaying slightly lower and more consistent values ranging from 2.15 to 2.25 nm (Figure 6c). These results suggest the overall compactness of protein after ligand binding, with only slight differences between docking-derived poses.

## 3 Discussion

The discovery of the carcinogenic potential of malachite green, a potent anti-saprolegniasis agent, rendered it unfit for use in aquaculture, resulting in the resurgence of saprolegniasis, a major cause of multimillion-dollar economic losses and food insecurity worldwide (National Toxicology Program, 2005). Therefore, novel and potent anti-saprolegnia drugs are urgently needed to support sustainable aquaculture and global food security.

Several alternative chemical and natural agents with reported anti-saprolegnia activity have been explored as replacements for malachite green, including linalool (Tang et al., 2024; Tang et al., 2025), chlorhexidine gluconate (Thakuria et al., 2022), potassium permanganate (Ackah et al., 2024), copper-ionophores-based formulations (Ogunwa et al., 2025), synthetic and natural essential oils (Bamal et al., 2018; Madrid et al., 2021; Mazloomi et al., 2022), curcumin (Tandel et al., 2021), silver nanoparticle-based formulations (Meneses et al., 2023), and various plant extracts (Mazloomi et al., 2022; Ackah et al., 2024; Madrid et al., 2024; Reda et al., 2024; Debnath, 2025; Dehkordi et al., 2025; Heydari and Firouzbakhsh, 2025). Our group recently implemented a non-targeted subtractive proteomics approach to identify suitable drug targets in *Saprolegnia*. Virtual screening against these targets led to the identification of several compounds with promising anti-saprolegnia activity (Kumar et al., 2020), however, their *in vivo* suitability remains to be verified. While this represents a valuable unbiased strategy, there is also a pressing need to complement it with a target-based approach. Species-specific inhibition of energy metabolism in Saprolegnia offers a promising and environmentally safer avenue. In particular, P450 fusion proteins are species-specific (Bamal et al., 2018) and represent attractive targets for structure-guided drug discovery. Taken together, integrating both approaches could accelerate the discovery of safe and effective therapeutics for sustainable aquaculture.

The current study focused on virtual screening targeting the *Saprolegnia parasitica* P450 fusion protein. Because its 3D structure is not available in the Protein Data Bank, we constructed a homology-modelled structure. Using this model, we performed screening with AutoDock, AutoDock Vina, and Dock6 and applied consensus scoring to rank resulting hits (Table 1).

Our virtual screening generated 100 top-ranked compounds. A subset was selected for *in vitro* tests based on binding score (Supplementary Table S1), commercial availability, and solubility. Additionally, these compounds were only tested up to a concentration of 2 mg/mL, which is quite reasonable from the perspective of their use in aquaculture for *in vitro* treatment. Only a small subset of the selected compounds demonstrated measurable activity against *Saprolegnia parasitica*. Notably, chlorhexidine (chlorhexidine gluconate) and diminazene exhibited strong and highly significant growth inhibition. Chlorhexidine was among the top-ranked hit compounds based on the cumulative score. In contrast, it was not the top hit compound in the cases of AutoDock and AutoDock Vina, which signifies our approach of implementing various independent docking tools when performing virtual drug screening. The IC_50_ of chlorhexidine was 11.87 μg/mL, which is significantly lower than that of many recently reported anti-saprolegnia compounds, including acetohydroxamic acid (MIC = 800 μg/mL), and is comparable to that of triclosan (MIC = 6 μg/mL) (Kumar et al., 2020). In a parallel investigation, chlorhexidine was predicted to bind SpHtp1, TKL kinase, and plasma membrane ATPase (Thakuria et al., 2022). Thus, its multi-target inhibition may contribute to its strong potency, supporting its potential as a promising anti-saprolegnia compound. In addition, chlorhexidine is well known for its antimicrobial and antifungal activity (Karpiński and Szkaradkiewicz, 2015). Its established safety in topical applications (≤2%) suggests a favorable basis for toxicity assessment in aquaculture (Xue et al., 2011), and it has been safely applied at concentrations up to 5% in surgical settings (Wade et al., 2021). It is also used as a reference anti-amoebic drug to combat *Acanthamoeba* infections. Due to its substantial surface-active and membrane-disrupting effects, it impedes pathogen structural integrity and metabolic activity. Its effectiveness is linked to interference with key enzymes, such as cytochrome P450 monooxygenases, which are crucial for the membrane stability and integrity, as well as sterol biosynthesis in microbial and parasitic cells (Abdel-Hakeem et al., 2024).

Diminazene is a well-known antiprotozoal drug used to treat babesiosis, leishmaniasis, and trypanosomiasis. It also exhibits antibacterial effects, including activity against Shiga toxin-producing *Escherichia coli* and inhibition of the transcriptional regulator QacR in *Staphylococcus aureus* (Wu et al., 2016). In humans, reported protein targets include PRDX5, AOC1, and PRSS1 (Wu et al., 2016; Rajapaksha et al., 2018), and it has demonstrated protective effects in models of liver injury, biliary fibrosis, cardiac ischemia/reperfusion injury, and renal fibrosis (Coutinho et al., 2022). In our study, diminazene displayed anti-saprolegnia activity with an IC₅₀ of 417 μg/mL, comparable to values reported for other recently identified candidates (Kumar et al., 2020). Although some toxicities have been reported, particularly neurological and hematological effects, these were not observed in rat blood cells at tested doses (Oliveira and Silva, 2021). Overall, chlorhexidine and diminazene exhibited significant *in vitro* activity, supporting their potential for further development as treatments for saprolegniasis.

The chemical space accessible for drug discovery is extremely large, with compound libraries such as GSK’s XXL, GDB17, and Enamine REAL containing approximately 10^12^–10^23^ compounds. High-throughput experimental screening on this scale is impractical. Thus, computational strategies have become essential to reduce candidate numbers from millions to a manageably few for laboratory testing. Exascale computing combined with highly parallelized virtual screening tools enables evaluation of ultra-large libraries (Murugan et al., 2022). However, success depends strongly on the accuracy of scoring functions. By using consensus scoring across three different docking programs, we increased confidence in the prioritization of candidate inhibitors. Such a virtual screening using multiple docking approaches and consensus scoring has been reported to be superior in the literature (Blanes-Mira et al., 2023; Manelfi et al., 2021; Rodrigo et al., 2021). Further they use various docking softwares such as the ones used in the current work and other softwares such as Glide, Smina, LeDock and rDock. Certain studies use the sum of scoring functions to rank the compounds as we followed in the current work but more studies need to be conducted to establish the correct way of doing consensus scoring.

From 100 top-ranked hits, eight compounds were selected for experimental validation based on commercial availability and solubility. Two of these demonstrated potent anti-saprolegnia activity, a 25% hit rate. In comparison, high-throughput screening campaigns in the biopharmaceutical industry typically identify one successful drug candidate from ~10,000 compounds (~0.01% success rate). Therefore, virtual screening followed by focused experimental testing offers a pragmatic and cost-effective strategy for identifying novel therapeutics. Molecular dynamics simulations (MDS) provided additional support by confirming the stability of ligand–protein complexes. Diminazene and chlorhexidine both formed stable complexes with the CYP5619A1 fusion protein (Filipe and Loura, 2022; Murugan et al., 2022). RMSD analyses showed that AutoDock-derived poses were the most stable for both compounds, while Vina poses displayed more pronounced fluctuations, consistent with previous findings that different docking tools can predict binding orientations with varying dynamic reliability (Feher, 2006; Houston and Walkinshaw, 2013). RMSF profiles revealed that most residues within the protein–ligand complexes remained stable, with flexibility primarily restricted to loop and N-terminal regions. Reduced mobility of catalytic pocket residues upon ligand binding suggested localized stabilization associated with effective P450 inhibition (Kelly and Kelly, 2013). Radius of gyration (Rg) results further indicated that the compactness of the protein was preserved, with slightly tighter packing observed in chlorhexidine-bound complexes relative to diminazene.

These computational results agree with the *in vitro* findings: chlorhexidine exhibited a substantially lower IC₅₀ than diminazene, confirming its higher potency. When compared to malachite green, an efficient but carcinogenic anti-saprolegnia agent (Green, 2004), chlorhexidine provides comparable stabilization of the CYP5619A1 active site with a more favorable toxicity profile (Karpiński and Szkaradkiewicz, 2015; Thakuria et al., 2022). Although chlorhexidine is less potent than malachite green, its low IC₅₀ and favorable safety profile suggest it may serve as a practical alternative rather than a direct potency-equivalent replacement. However, *in vivo* studies and formulation optimization will be required to determine its efficacy and safety under realistic aquaculture conditions. In contrast, diminazene was less potent but has broad antiparasitic properties (Rajapaksha et al., 2018), and its moderate binding stability suggests potential for structure-based optimization.

Together, these results demonstrate that integrating consensus docking, MD-based stability analysis, and experimental validation constitutes a robust pipeline for rational discovery of anti-saprolegnia therapeutics (Bamal et al., 2018). This workflow is well-suited to efficiently explore the vast chemical space and accelerate the development of safer and more effective treatments for saprolegniasis.

## 4 Materials and methods

### 4.1 Ligand preparation

The FDA-approved drug data set containing 8,773 compounds was extracted from DrugBank (Wishart et al., 2006) database. The ligand (drug) molecules were prepared by using Openbabel (O'Boyle et al., 2011) software to convert two-dimensional (2D) into three-dimensional (3D) structures. The hydrogen atoms were added to the ligand structures, and Gasteiger charges were added (using prepare_ligand4.py program which is part of the AutodockTools) before the molecular docking for each ligand. The sampling was performed over the translational, rotational, and torsional degrees of freedom of the ligands within the protein binding site to determine the most stable binding mode of the ligand. Different scoring functions are adopted in the three software used in this work: AutoDock4.0, AutoDock Vina and Dock6. Numerous conformations for each ligand are generated and ranked using the scoring function. Malachite green was used as a control compound for P450 in these ligands.

The Modeller software (Sali, 1995) was used to build a 3D model of the CYP5619A1’s P450 domain. Among all templates, CYP120A1 (PDB ID: 2VE3) (Kühnel et al., 2008) showed the lowest E-value and was therefore selected as the template to build the 3D model of CYP5619A1. The modelled structure is shown in Figure 1. The HEME group has been considered during the modelling of the structure. The modelled CYP5619A1’s P450 domain’s heme group parameters were generated using the MCPB.py (Li and Merz, 2016) program, and minimisation was carried out through Amber 16 using the Amber ff19SB (James, 2007; Tian et al., 2019) force field at 300 K. An explicit solvent model TIP3P water (Mark and Nilsson, 2001) was used, and the complexes were solvated with a 10 Å water cap. The ions were added as counterions to neutralise the system. The protein was then minimised through 5,000 steps of Steepest Descent (SD) followed by Conjugate Gradient (CG). The resulting minimised structure was selected for molecular docking studies.

### 4.2 Molecular docking

The virtual screening calculations were carried out using three different molecular docking software packages: AutoDock (Huey et al., 2007; Morris et al., 2009), AutoDock Vina (Trott and Olson, 2010) and Dock6 (Allen et al., 2015; Balius et al., 2024) for the P450 target. The drug compounds were chosen from the DrugBank database (Wishart et al., 2018). A cumulative score (Score3) was designed for all compounds docked using these three docking tools. The compounds were ranked based on Score3 to prioritise them for experimental validation of these hits.

### 4.3 Dock6

The molecular surface of the binding site of the P450 domain was generated by the *dms* program with a probe radius of 1.4 Å. The *sphgen* program calculated the spheres over the entire surface with default settings from the binding site residue. A rectangular box was defined to determine the docking site based on selected sphere clusters using the *showbox* program. The energy scoring values were calculated using a *grid* program on all grids and subsequently screened using the *Dock6.mpi* program to perform docking simulations. During each docking, both the receptor and ligand were kept rigid. The ligand was positioned using the automated matching algorithm with a maximum of 1,000 orientations. The numerically highest interaction energy (IE) and the corresponding conformation were recorded. All the ligands were ranked based on their interaction energy values, and molecules with the highest IE were taken as top hits.

### 4.4 AutoDock and AutoDock Vina

The AutoDock 4.2 was used to dock the DrugBank compounds with the P450 domain. PDBQT files were generated using AutoDock Tools for both the DrugBank compounds and the macromolecule for docking purposes. AutoGrid was used to generate a grid box for docking. The grid box dimensions were chosen as 66 × 66 × 96, with a centre at 49.006, 41.256, 42.11, and a default grid spacing of 0.375 Å. AutoDock was then used to screen the compounds with the P450 domain. The Lamarckian Genetic Algorithm (LGA) was used to simulate with default parameters. For AutoDock Vina, similar grid parameters were considered, and screening was performed with the Vina package. In the case of Autodock Vina, the default parameters for exhaustiveness (value used was 8) which controls the sampling robustness and number of binding modes to be stored (10) were used. The default grid spacing in Autodock Vina is 1 Å and so a grid box size of 25 × 25 × 36 Å^3^ has been used.

The molecular docking results were visualized by using ChimeraX (Pettersen et al., 2021) and Discovery Studio (BIOVIA, 2021) to analyze and prepare the 2D/3D images of interactions between the ligands and the target protein.

### 4.5 Molecular dynamics simulation

Molecular dynamics (MD) simulations were performed to investigate the binding stability and binding behaviour of protein-ligand complexes over Time. MD simulations were carried out for the most stable docked complexes of the P450 protein with Diminazene and Chlorhexidine, which had the highest binding energies according to AutoDock, Dock6, and AutoDock Vina. For this, GROMACS version 2023 (Berendsen et al., 1995) was used with the OPLS-AA force field. The solvation of protein-ligand complexes was performed in a cubic box using the TIP3P water model, with counter-ions added to neutralise the system. The steepest descent algorithm was used to minimise energy, which was followed by equilibration in NVT (100 ps, 300 K) and NPT (100 ps, 1 bar) ensembles to maintain constant temperature and pressure. During the production phase, a simulation of 100 ns was performed with a time step of 2 fs. The Particle Mesh Ewald (PME) method treated long-range electrostatics, with non-bonded interactions limited to 1.0 nm. The LINCS algorithm was employed to control bond lengths, and periodic constraints were applied in all directions. Then, GROMACS utilities were used to perform trajectory analyses and calculate root mean square deviation (RMSD), root mean square fluctuation (RMSF), and radius of gyration (Rg) of the protein–ligand complexes generated from AutoDock, Dock6, and Vina docking poses for both Diminazene and Chlorhexidine.

### 4.6 Experimental screening of selected hits

The compounds from the virtual drug screening list were selected based on their affinity, cost, water solubility and availability. To validate the selected compounds *in vitro,* we performed a visual growth inhibition assay at various concentrations, ranging from 100 μg to 1 mg/mL of liquid culture medium, Machlis medium. The roughly equal slices of *S. parasitica* culture on PDA (potato dextrose agar) were added to 1 mL of culture medium 12 h before the application of the drug in a 12-well cell culture plate (Corning, USA). After 12 h, 1 mL of medium containing twice the desired drug concentration was added to each well for various concentrations, and 1 mL of medium without drug was added to the control well. The *S. parasitica* was grown in the drug-containing medium for 48 h, and the plate was visually screened for growth inhibition. These compounds, which showed growth-inhibiting potential in the initial screen, were selected for the MTT assay to determine their IC_50_.

### 4.7 MTT assay

To perform the MTT assay, sodium resazurin salt (Almar Blue) was used to measure the viability of the *S. parasitica* after drug treatment. Roughly equal slices of *Saprolegnia parasitica* culture on PDA, were added to 1 mL of Machlis’s culture medium in triplicate for each concentration and control. After 12 h. 100 μL of Almar Blue was added to the culture and incubated for 2 h. A 100 μL of culture medium containing the Almar Blue was sampled from the drug-treated and untreated Saprolegnia cultures. The resorufin produced by NADPH dehydrogenase activity from resazurin (O'Brien et al., 2000) was excited at 575 nm and the emitted fluorescence at 590 was measured using CLARIOstar Plus plate reader (BMG LabTech, Germany). The relative cell viability of each replicate was calculated as base viability due to variable inoculated cultures and was used to normalize growth variation across the plate. After washing the cultures, the Machlis culture medium was replaced with a drug-containing Machlis medium at various concentrations ranging from 0–1,000 ug/ml in each assay plate. These cultures with drugs were incubated at 25 °C for 48 h. Cell Viability was measured again as described above, after 48 h of drug treatment. It was normalised with base viability to remove variation due initial inoculums. After normalisation, the relative viability was used to measure the IC_50_ for each hit compound using GraphPad Prism software.

## Data Availability

The original contributions presented in the study are included in the article/Supplementary material, further inquiries can be directed to the corresponding authors.
